# Chemical Composition, Antifungal and Antitumor Properties of Ether Extracts of *Scapania verrucosa* Heeg. and its Endophytic Fungus *Chaetomium fusiforme*

**DOI:** 10.3390/molecules13092114

**Published:** 2008-09-04

**Authors:** Lei Guo, Jin-zhong Wu, Ting Han, Tong Cao, Khalid Rahman, Lu-ping Qin

**Affiliations:** 1 Department of Pharmacognosy, School of Pharmacy, Second Military Medical University, Shanghai 200433, P.R. China; 2 Academy of integrative medicine, Fujian University of Traditional Chinese Medicine, Fuzhou, Fujian 350003, P.R. China; E-mail: jinzhongfj@126.com; 3 Department of Biology, College of Life and Environment Sciences, Shanghai Normal University, Shanghai 200234, P.R. China; E-mail: CT1946@263.net; 4 Faculty of Science, School of Biomolecular Sciences, Liverpool John Moores University, Byrom Street, Liverpool L3 3AF, England, UK

**Keywords:** *Scapania verrucosa*, *Chaetomium fusiforme*, GC/MS, antifungal activity, antitumor activity.

## Abstract

An endophytic fungus *Chaetomium fusiforme* was obtained from a liverwort, *Scapania verrucosa*. A comparison of the constituents of the ether extracts between *S. verrucosa* and the *C. fusiforme* culture was investigated by gas chromatography-mass spectrometry (GC/MS). The yield of ether extract based on dried plant material was 0.6% and 59 compounds were found in *S. verrucosa*. (+)-Aromadendrene (9.12%), hexadecanoic acid (6.92%), 6-isopropenyl-4,8a-dimethyl-1,2,3,5,6,7,8,8a-octahydro-naphthalen-2-ol (5.97%), *s*-tetrachloroethane (5.61%) and acetic acid (5.30%) were found to be the most abundant components among the 49 characterized compounds in *S. verrucosa*, which represented 84.64% of the total extract. However, the constituents of the cultured endophyte extract contained mainly acetic acid (35.05%), valeric acid, 3-methyl-, methyl ester (21.25%), and butane-2, 3-diol (12.24%). Although the extracts of *S. verrucosa* and its endophyte showed little chemical composition correlation, both of them demonstrated antifungal and antitumor activities. Furthermore, *C. fusiforme* has displayed a wider range of antimicrobial and antitumor activities, which were better than the host plant. These results could support the suggestion of endophytes as an alternative of the host for medicinal activity.

## Introduction

Bryophytes are known to possess various rare and novel natural products. Many of these exhibit interesting biological activities such as antimicrobial, cytotoxic, insect antifeedant, muscle relaxing, some enzyme inhibitory, apoptosis inducing activity, lipoxygenase, calmodulin, hyaluronidase, cyclooxygenase, thrombin inhibitory activity and neuritic sprouting activity [1,2,3,4,5]. Liverworts contain oil bodies which are composed of a large amount of mono-, sesqui- and diterpenoids, as well as aromatic compounds [6,7]. In particular, liverworts contain pinguisane-type sesquiterpenoids, sacculatane-type diterpenoids and bis (bi-benzyl) aromatic compounds which have not been found in higher plants [2,3,4,5,6,7,8].

*Scapania verrucosa* Heeg. (Scapaniaceae), is a liverwort, mainly found distributed in south-central China, Nepal and the Himalayan region of Jammu and Kashmir. It is about 10–25 mm tall and grows on forest ground, rocks and decaying wood [9,35]. The plants from Scapaniaceae, such as *S. aequiloba, S. ampliata, S. aspera, S. bolandeli, S. nemorea, S. ornithopodiodes, S. paludosa, S. parvitexta, S. stephanii, S. subalpina, S. uliginosa,* and *S. undulate* were chemically investigated previously [7,10,11,12,13,14,15].

Endophyte, by definition, is one which resides in the tissues beneath the epidermal cell layers and causes no apparent harm to the host [16]. Recent studies have shown that fungal endophytes are ubiquitous in plant species [17,18] and are mutualistic to their host. At least some of them are thought to be receiving nutrition from the plant in exchange for producing special substances such as secondary metabolites to protect the host from successful attack by fungi, pests and mammals. In support of this idea, metabolites of endophytes have been reported to inhibit a number of micro-organisms [19,20]. On one hand, endophytes can produce similar or the same biologically active constituents as its host, such as an endophytic fungus producing taxol [21]. On the other hand, fungi are a prolific source of metabolites with significant biological activities. Many important anticancer, antifungal and antibacterial chemotherapeutics are either microbial metabolites or their semisynthetic derivatives. Investigating the metabolites of endophytic fungi can increase the chance of finding novel compounds. An intensifying stream of attention is being directed to the endophytes from unique habitats living in special environments [22,23]. 

The difficulty of acquiring large amounts of liverworts is due to the fact that many of them are spread over wide areas and occur only in small populations. A promising way out of this impasse is the use of endophytes of liverworts. The endophytes could be utilized for their fermentation and biotechnology capabilities as an alternative mode of production of the bioactive components. Distinctly from plants, endophytes can be cultured quickly and the biomass can be accumulated by large scale fermentation. 

To the best of our knowledge, there are no reports on *S. verrucosa* extracts with antimicrobial or antitumor activity. In this paper, a comparison of the chemical components between *S. verrucosa* and the culture of its endophyte was carried out. This study also reported the antimicrobial and antitumor activities of the essential oil from *S. verrucosa* and its endophyte *Chaetomium fusiforme*. 

## Results and Discussion

### Chemical components of the plant and the endophyte extracts

The yield of pale green colored ether extract of *S. verrucosa* was about 0.6%. The endophyte oil was yellow one. The components identified from *S. verrucosa* extract, their retention indexes and their percentage composition are summarized in Table 1. The results of the GC/MS analysis of the composition of the endophytic fungus and blank ferment broth PDB are presented in Table 2.

**Table 1:** Chemical composition of *S. verrucosa* extract.

Compounds	RI^a^	Content/%
n-dodecane	1200	0.22
2-heptenal	1334	0.18
3-octanol, acetate	1344	0.19
octen-1-ol, acetate	1385	2.84
n-tetradecane	1400	0.29
acetic acid	1425	5.30
aminic acid	1470	0.87
s-tetrachloroethane	1516	5.61
β-bourbonene	1528	1.83
aromadendrene, dehydro-	1541	0.42
(-)-aristolene	1582	2.06
β-elemen	1599	1.30
calarene	1604	5.01
cedr-8(15)-ene	1609	1.05
1H-cyclopropa[a]naphthalene, decahydro-1,1,3a-trimethyl-7-methylene	1614	2.74
9-methyltetracyclo[7.3.1.0(2.7).1(7.11)]tetradecane	1655	4.49
(+)-aromadendrene	1660	9.12
β-farnesene	1672	0.51
acroadiene	1689	0.34
n-heptadecane	1700	0.26
thujopsene-(12)	1715	0.19
eremophila-1(10), 11-diene	1732	0.14
c-neoclovene	1747	0.12
11-isopropylidenetricyclo[4.3.1.1(2,5)]undec-3-en-10-one	1784	0.09
n-octadecane	1800	0.18
(E,E)-2,4-decadienal	1821	0.16
(+)-cuparene	1838	0.13
hexanoic acid	1851	0.15
[2,4,4-trimethyl-1-(2-methylpropanoyloxy)pentan-3-yl] 2-methylpropanoate	1884	0.17
phytol	1929	1.76
cyclohexene, 6-(2-butenyl)-1,5,5-trimethyl-(E)-	2027	0.47
ledol	2095	0.19
2-(4a,8-dimethyl-1,2,3,4,4a,5,6,7-octahydro-naphthalen-2-yl)-prop-2-en-1-ol	2124	0.67
spathulenol	2135	4.74
guaia-1(5),11-diene	2162	0.09
thujopsene	2182	0.37
6-isopropenyl-4,8a-dimethyl-1,2,3,5,6,7,8,8a-octahydro-naphthalen-2-ol	2241	5.97
aromadendrene oxide-(2)	2299	1.22
cyclohexene, 6-(2-butenylidene)-1,5,5-trimethyl-(Z,E)-	2323	0.47
(-)-spathulenol	2464	0.41
7-acetyl-2-methyl-5-isopropylbicyclo[4.3.0]nonane	2524	1.61
thunbergol	2538	0.65
tetradecanoic acid	2585	0.25
platambin	2686	2.22
hexadecanoic acid	2756	6.92
4,8,13-duvatriene-1,3-diol	2955	3.72
stearic acid	3020	1.82
9-octadecenoic acid (Z)-	3072	1.24
linoleic acid	3170	3.92
Total identified		84.64

^a^ calculated from retention times relative to n-alkanes under the same GC conditions (DB-Wax column).

**Table 2:** A comparative analysis of the composition of *Chaetomium fusiforme* and the ferment broth PDB without inoculation.

*Chaetomium fusiforme*			PDB^a^		
**Compounds**	**RI^b^**	**Content/%**	**Compounds**	**RI^ b^**	**Content/%**
butyl alcohol	1195	1.35	2-n-pentylfuran	1259	0.24
methyl 2-oxopropionate	1276	0.14	ethyl pyroracemate	1303	0.15
acetoin	1314	1.70	devoton	1347	0.90
1,2-propanediol	1336	0.21	ethyl vinyl ketone	1414	0.51
3-octanol	1384	1.61	acetic formic anhydride	1436	10.45
acetic acid	1425	35.05	3-furancarboxaldehyde	1455	1.29
2-furancarboxaldehyde	1443	0.28	aminic acid	1470	2.39
aminic acid	1470	0.56	propionic acid	1493	0.36
butane-2,3-diol	1485	11.24	2-butyl-1-octanol	1528	0.54
dimethylsuccinate	1526	0.61	2-cyclopentene-1,4-dione	1535	0.25
butyrolactone	1561	0.23	acrylic acid	1560	0.28
acetophenone	1573	0.26	furfuryl alcohol	1575	2.66
4-penten-1-ol,2,2,4-trimethyl-	1592	0.38	2-furancarboxylic acid, tetrahydro-3-methyl-5-oxo-	1581	0.67
5-hexalactone	1669	5.71	n-heptanoic acid	1623	0.27
valeric acid,3-methyl-,methyl ester	1677	21.25	2-ethylcyclohexanone	1651	0.57
ethyl 3-hydroxy-2,2-dimethylbutanoate	1699	0.29	heptadecane,2,6,10,15-tetramethyl-	1660	0.86
benzeneethanol	1734	6.17	n-caproic acid	1690	1.73
hydroxymethylfurfurole	1812	1.00	phenol, p-sec-butyl-	1701	0.44
p-cresol	1835	0.25	1,2-dimethylpropyl acetate	1713	0.69
o-cresol	1840	0.18	benzenemethanol	1717	0.24
methyl vinylcarbinol	1861	0.67	corylon	1768	0.30
benzeneacetic acid	1878	0.26	maltol	1777	2.47
methyl vinylcarbinol	1892	0.56	methyl 2-furoate	1808	0.86
methyl phenylglycalate	1923	0.26	hydroxymethylfurfurole	1812	20.79
monomethyl succinate	1988	2.66	pyrrole-2-carboxaldehyde	1814	1.26
2-furoic acid	2048	0.43	pantolactone	1818	0.76
pyrrolidine-5-one, 2-[3 -hydroxypropyl]-	2167	0.25	benzeneacetic acid	1878	5.68
4-(2-hydroxyethyl)phenol	2565	2.37	2-methoxy-4-vinylphenol	1915	0.69
total		95.94	pyranone	1962	8.35
			3,5-dihydroxy-2-methyl-4-pyrone	1969	1.08
			hemineurine	1980	2.62
			3-hydroxypyridine	2048	3.11
			o-aminophenol	2054	2.50
			butanimide	2081	1.96
			hexadecanoic acid	2476	1.05
			niacinamide	2549	2.61
			palmitamide	2858	1.89
			cyclo(leucyloproly)	2870	7.81
			total		91.27

^a^ Potato Dextrose Broth ^b^ calculated from retention times relative to n-alkanes under the same GC conditions (DB-Wax column).

Fifty-nine components were detected in the ether extract of *S. verrucosa*, of which 49 components representing 84.64% were identified. According to our results, the main constituents of the extract from *S. verrucosa* were aromadendrene (9.12%), hexadecanoic acid (6.92%), 6-isopropenyl-4,8a-dimethyl-1,2,3,5,6,7,8,8a-octahydronaphthalen-2-ol (5.97), *s*-tetrachloroethane (5.61%), and acetic acid (5.30%). Finally, there were ten other volatile chemical constituents that remained unidentified since spectral data obtained did not appropriately match any compound in the NIST database.

The genus *Scapania* produces many kinds of sesquiterpenoids and diterpenoids which are ubiquitous in other liverworts. The most common sesquiterpenes are anastreptene and aromadendrane hydrocarbons. The European *S. undulata* mainly comprises longifolene-type, longiborneoltype, (+)-ent-*epi*-cubenol-type, and labdane-type sesquiterpene [11]. *S. undulata* collected in Belgian elaborates muurolane-type sesquiterpenoids [12,24]. The major components of the Japanese *S. undulata* are (-)-longiborneol and a-longipinene. In addition, it contains longipinanol, labdanes and dimeric labdanes [10]. The predominant components of *S. nemorea* in France and Germany are *cis*-clerodanes and a secoclerodane-type diterpenoid [14]. Another German specimen produces diplophyllolide and a secoclerodane [25,26]. *S. bolanderi* elaborates not only verrucosane and neoverrucosane diterpenoids [27] but also trans-clerodane-type diterpenoid [13]. *S. subalpina* and *S. uliginosa* both elaborate the same sesquiterpene hydrocarbons, longifolene, and isolongifolene. In sum, aromadendranes, clerodanes, labdanes, longibornades, longifolanes, longipinanes, and muurolanes are very valuable chemical markers of the genus *Scapania* [28]. Comparison with previous study of the chemical composition of related species, *S. verrucosa* also produces a great amount of aromadendrane. However, our investigation showed different qualitative profiles. Calarene, maaliane-type and aristolane-type sesquiterpene, which appear in our study as abundant constituents, seem to be in traces or absent in other species of the genus *Scapania*.

In the analysis of the ether extract obtained from the culture of *Chaetomium fusiforme*, there are 40 compounds were found. The number of identified compounds was 28, which represented 95.94%, while the number in blank broth PDB is 38, representing 91.27%. As for *C. fusiforme,* acetic acid (35.05%), valeric acid, 3-methyl-, methyl ester (21.25%) and butane-2,3-diol (12.24%) were characterized as the dominating compounds. In the extract of blank culture medium, hydroxymethylfurfurole (20.79%), acetic formic anhydride (10.45%), pyranone (8.35%), cyclo (leucyloproly) (7.81%), and benzeneacetic acid (5.68%) are the most abundant.

It is apparent from the data shown that the components of *S. verrucosa* were predominantly mono- and sesquiterpenes, dominated by aromadendrene, whilst its endophytic fungus *C. fusiforme* seldom produced sesquiterpenes. Interestingly, both of them produced not quite abundant acetic acid and aminic acid, which were known to kill a wide spectrum of fungi and bacteria. Our analysis of the culture of *C. fusiforme* and the broth without inoculation showed nearly no correlation in their chemical composition and there are just hydroxymethylfurfurole and benzeneacetic acid in common. From this, we conclude that the fungus could consume and transfer the composition of the culture medium to produce versatile metabolites. 

### Antifungal activity

The antifungal activity of the ether extracts from *S. verrucosa* and *C. fusiforme* against the microorganisms considered in the present study was quantitatively assessed (Table 3).

**Table 3:** Antimicrobial activity of the ether extract of *S. verrucosa* and *C. fusiforme*.

IC_80_(μg/mL)			
Test fungi	* S. verrucosa*	*C. fusiforme*	KCZ^a^
*Candida albicans* ATCC76615	32	32	0.0625
*Cryptococcus neoformans* ATCC32609	64	32	0.0625
*Trichophyton rubrum*	64	ND^b^	0.25
*Aspergillus fumigatus*	8	32	1
*Pycricularia oryzae*	>128	128	8

^a^ Ketoconazole as positive control ^b^ Not determined.

The plant extract displayed broader antimicrobial spectrum and stronger toxicity to most of the tested microbes. But the extract of this endophytic fungus cultures was more toxic to the growth of *Cryptococcus neoformans*. 

### Antitumor activity

The screening of antitumor activity was also conducted, and the results are represented in Table 4.

**Table 4:** Antitumor activity of the ether extract of *S. verrucosa* and *C. fusiforme*.

IC_50_(μg/mL)			
Cell line	*S.vercossa*	*C. fusiforme*	DOX ^a^
A549	>100	>100	0.0207
LOVO	>100	9.11	0.734
HL-60	42.92	4.06	0.00190
QGY	90.78	31.23	0.0110

^a^ Doxorubicin as positive control

The host plant was moderately inhibitory to all test tumor cells when compared with DOX. The fungus isolated from *S. verrucosa* possessed much better cytotoxic activities than the host plant with most of IC_50_ values being below 50 μg/mL. 

Many antibiotics have encountered drug resistance or caused severe adverse drug reactions, and there is an urgent need to search for new antibiotics. Several recent studies have reported the isolation of novel compounds with antimicrobial activity from the cultured endophytic fungi [29,30]. Others have also reported the isolation of endophytic fungi with anti-microbial, anti-cancer and anti-malarial activities from Thai medicinal plants [31,32,33,34]. 

The present study shows that endophytic fungi isolated from *S.verrucosa* also have a wide range of antifungal and antitumor activities, which were not strong, but better than the host plant, although the vast majority of their chemical constituents were not the same. Interestingly we have hereby disclosed a microbial source of the important phytochemical succedaneum.

Thus, a smart manipulation of the pathway would lead to a scaled-up fermentation production of some important bioactive compounds. This seems more workable than the desired microbial production of taxol, which is still out of sight although, its producing endophyte has been reported a decade ago [21]. In conclusion, the endophytic fungus of *S. verrucosa* is a versatile producer of the variously bioactive metabolites. 

Because of the diversity and complexity of the natural mixtures of bioactive compounds in the crude plant extract and fungal cultures, it is rather difficult to characterize every compound present and elucidate its structure in a single study. According to the information presented, the compounds considered characteristic of *C. fusiforme* are produced by the microorganisms, at least in the selected culture medium. If cultured in other conditions, such as different cultivation time and water content, very different compounds could be produced. However, further investigation is still needed to discover the unidentified/ unknown bioactive constituents in the endophytic fungal isolates and its host. 

## Experimental

### Plant Material and Prepare for the extract

Samples of *S. verrucosa* were collected from Yandang Mountain, Zhejiang Province, P.R. China, in November, 2006. Taxonomic identification [35] was performed by Professor Caotong from Shanghai Normal University, Shanghai, China. Voucher specimens of *S. verrucosa* have been deposited at the Herbarium of Department of Pharmacognosy, Second Military Medical University, Shanghai, P.R. China. Plant material was air-dried in the shade prior to extraction. The samples (10 g) were soaked with diethyl ether (100 mL) at room temperature. After 24 h, the mixture was filtered through Whatman filter paper using a Buchner funnel. The solvent was removed with a rotary vacuum evaporator at 40°C. This procedure was repeated three times. The volatile oil yield was 0.6% and it was stored in dark vials at 4°C before analyses [percentage extract yield (w/w) was estimated as extract weight/starting material weight x 100].

### Isolation of strains

The endophytic fungal strain 37 was separated from the *S. verrucosa* according to the procedure described by Schulz *et al*. [36]. Specifically, *S. verrucosa* was washed with distilled water, sterilized with 75% ethanol for 1 min and 2.5% sodium hypochlorite for 15 min, then rinsed in sterile water for three times. Both borders of sterilized segments were cut off, and the rest was incubated at 28±1 °C on PDA medium supplemented with ampicillin (200 μg/mL) and streptomycin (200 μg/mL) to inhibit the bacterial growth until the mycelium or colony originated from the injury surface. The mycelium was purified and cultured under the same conditions. Another segment of the same origin without surface sterilization was cultured as a negative control to check the presence of contaminated microbes on the segment surface. The purified endophytic fungi were numbered and transferred to fresh PDA (Potato Dextrose Agar) slants separately and were kept at 4 °C after being cultured at 28 ± 1 °C for 7 days.

### Fermentation and extraction of the endophytic fungus

The inoculum was prepared by transferring the periphery of 7-day-old Petri dish cultures of the endophyte into 250 mL flasks containing broth (100 mL; potato: 200 g, dextrose: 20 g, H_2_O: 1000 mL), followed by shaking (280 rpm) continuously for 7 days at 28±1 °C. The ferment broth of the endophyte was filtered. The filtrate was extracted with diethyl ether and the obtained extracts were concentrated in a rotatory evaporator at 45 °C. In order to ascertain whether any of the isolates obtained in this study was the constituent of the PDB extract in the substrate, the EtOAc extract of the sterile medium treated equally but without inoculation of the microorganism was subjected to an GC–MS comparison showing that all the isolates were indeed produced by the title endophytic fungus.

### Essential oil analysis

Chromatography was performed on a DB-Wax capillary column (30 m x 0.25 mm ID and 0.25 μm film thickness). The electron impact technique (70 eV) was used. The carrier gas was helium at a flow rate of 1.0 mL/min, and 1 μL of sample was injected. The injector and detector temperatures were 230 °C and 200 °C respectively. The column oven was programmed as follows: initial temperature: 60 °C; initial time 2.0 min; program rate 10 °C /min; final temperature 250 °C; final time 10 min. The sample was dissolved in CH_2_Cl_2_ and a split injection technique was used. The identification of the compounds was based on comparison of their retention indexes (RI), obtained using n-alkanes (C11-C31), and retention time. They were also confirmed by comparison of their mass spectra with the NIST/NBS-Wiley library spectra and literature data. Relative percentage amounts were calculated from TIC by the computer.

### Identification of the endophyte

The endophytic fungal strain was identified by the morphological method. The morphological examination was performed by scrutinizing the fungal culture, the mechanism of spore production, and the characteristics of the spores. All experiments and observations were repeated at least twice. The strain was identified as *C. fusiforme* by the Institute of Microbiology, Chinese Academy of Sciences, Beijing, China. 

### Antifungal activity test

The essential oil of the host and its endophyte were screened for antifungal activity against *Candida albicans, Cryptococcus neoformans, Trichophyton rubrum, Aspergillus fumigatus, Pycricularia oryzae*. IC_80_ were determined using the modified liquid dilution method performed in 96 well micro-trays. The antifungal activities of the essential oil are reported in Table 3 and the values presented are an average of triplicate. Sabouraud Dextrose Agar was employed for fungal growth. Dilutions of *S. Verrucosa* and its cultured endophyte extract were prepared in dimethyl sulfoxide (DMSO). The extract solutions were serially diluted (4:1) in 96-well plates. Organisms at a concentration of approximately 1-5×10^3^ colony forming units (CFU/ml) were then added to each well. Plates were made in triplicate and incubated at 35 ^◦^C for about 24 h for *Monilia*, about 72 h for *Cryptococcus* and about 168 h for hyphomycete and their turbidity obtained by measuring optical density at 630 nm. Test substance concentrations at which fungus proliferation was reduced by 80% are given as IC_80_ values. The standard antifungal agent ketoconazole was used as a positive control and experiments were repeated at least three times. 

### Antitumor activity test

Test cells were grown in RPMI 1640 including 100 units/mL penicillin and 100 μg/ml streptomycin supplemented with 15% new-born bovine serum (NBS) at 37 °C in a 5% CO_2_ atmosphere. For experimentation, the exponentially growing cells (4~6×10^4^) were used. Then, cells were incubated in the presence of 1000 μg/mL samples in DMSO for 72 h at 37°C. After removal of the sample solution and washing with phosphate- buffered saline (pH7.4), 10 μl/well of 0.5% 3-(4,5-dimethyl-2-thiazolyl)-2,5-diphenyl-2H-tetrazolium bromide cells (MTT) phosphate-buffered saline solution was added. After a further 4 h of incubation, 0.04 M HCl was added. Viable cells were determined by measuring the absorbance at 570 nm. Measurements were performed 3 times, and the concentration required for a 50% inhibition of viability (IC_50_) was determined graphically.

## References

[1] Frahm J.P., Kirchhoff K. (2002). Antifeeding effects of bryophyte extracts from *Neckera crispa* and *Porella obtusata* against slug *Arion lusitanicus*. Cryptogamie, Bryologie.

[2] Asakawa Y., Atta-ur-Rahman (1988). Biologically active substances found in Hepaticae. Studies in Natural Products Chemistry.

[3] Asakawa Y., Chopra R.N., Bhatla S.C. (1990). Biologically active substances from bryophytes. Bryophytes Development: Physiology and Biochemistry.

[4] Asakawa Y., Zinsmeister H.D., Mues R. (1990). Terpenoids and aromatic compounds with pharmacological activity from bryophytes. Bryophytes: Their Chemistry and Chemical Taxonomy.

[5] Asakawa Y., Herz W., Kirby W.B., Moore R.E., Steglich W., Tamm C. (1995). Chemical constituents of the bryophytes. Progress in the Chemistry of Organic Natural Products.

[6] Nagashima F., Nondo M., Uematsu T., Nishiyama A., Sato S., Sato M., Asakawa Y. (2002). Cytotoxic and apoptosis-inducing ent-kaurane-type diterpenoids from the Japanese liverwort Jungermannia truncata Nees. Chem. Pharm. Bull..

[7] Asakawa Y., Herz W., Grisebach H., Kirby G.W. (1982). Chemical constituents of the Hepaticae. Progress in the Chemistry of Organic Natural Products.

[8] Asakawa Y., Romeo J.T. (1999). Phytochemistry of Bryophytes. Biologically Active Terpenoids and Aromatic Compounds from Liverworts. Phytochemicals in Human Health Protection, Nutrition, and Defense.

[9] Lars S., Ana S., Mariana S. (2007). Rarity patterns in members of the Lophoziaceae/Scapaniaceae complex occurring North of the Tropics – Implications for conservation. Biol. Conserv..

[10] Yoshida T., Toyota M., Asakawa Y. (1997). Scapaundulins A and B, two novel dimeric labdane diterpenoids, and related compounds from the Japanese liverwort *Scapania undulata* (L) Dum. Tetrahedron Lett..

[11] Adioa A.M., Paulb C., Klotha P., Königa W.A. (2004). Sesquiterpenes of the liverwort *Scapania undulata*. Phytochemistry.

[12] Nagashima F., Asakawa Y. (2001). Sesqui- and diterpenoids from two Japanese and three European liverworts. Phytochemistry.

[13] Tazakia H., Hayashida T., Furuki T., Nabeta K. (1999). Terpenoid from the liverwort *Scapania bolandeli*. Phytochemistry.

[14] Geis W., Buschauer B., Becker H. (1999). cis-Clerodandes from axenic cultures of the liverwort *Scapania nemorea*. Phytochemistry.

[15] Mues R., Huneck S., Connolly J.D., Rycroft D.S. (1988). capaniapyrone-A, a novel aromatic constituent of the liverwort *Scapania undulata*. Tetrahedron Lett..

[16] Stone J.K., Bacon C.W., White J.F., Bacon C.W., White J.F. (2000). An Overview of Endophytic Microbes: Endophytism Defined in Microbial Endophytes.

[17] Faeth S.H., Hammon K.E. (1997). Fungal endophytes in oak trees: long-term patterns of abundance and association with leafminers. Ecology.

[18] Huang W.Y., Cai Y.Z., Xing J., Corke H., Sun M. (2007). Potential antioxidant resource: endophytic fungi isolated from traditional Chinese medicinal plants. Econ Bot..

[19] Fisher P.J., Anson A.E., Petrini O. (1984). Antibiotic activity of some endophytic fungi from ericaceous plants. Bot. Helv..

[20] Gurney K.A., Mantle P.G. (1993). Biosynthesis of 1-N-methylalbonoursin by an endophytic S*treptomyces sp*. isolated from perennial *ryegra*ss. J. Nat. Prod. (Lloydia).

[21] Stierle A., Strobel G., Stierle D. (1993). Taxol and taxane production by *Taxomyces andreanae*, an endophytic fungus of Pacific Yew. Science.

[22] Tan R.X., Zou W.X. (2001). Endophytes: a rich source of functional metabolites. Nat. Prod. Rep..

[23] Hawksworth D.C., Rossman A.Y. (1987). Where are the undescribed fungi. Phytopathology.

[24] Nagashima F., Suda K., Asakawa Y. (1994). Cadinane-type sesquiterpenoids from the liverwort *Scapania undulata*. Phytochemistry.

[25] Asakawa Y., Inoue H., Toyota M., Takemoto T. (1980). Sesqui- terpenoids of fourteen *Plagiochila* species. Phytochemistry.

[26] Ohta Y., Andersen N.H., Liu C.B. (1977). Sesquiterpene con-stituents of two liverworts of genus *Diplophyllum*, Novel eudesmanolides and cytotoxicity studies for enantiomeric methylene lactones. Tetrahedron.

[27] Matsuo A., Atsumi K., Nakayama M. (1984). Isolation of seven verrucosane diterpenoids from the liverwort *Scapania bolanderi*. Z. Naturforsch..

[28] Asakawa Y. (2004). Chemosystematics of the Hepaticae. Phytochemistry.

[29] Liu J.Y., Liu C.H., Zou W.X., Tan R.X. (2003). Leptosphaeric acid, a metabolite with a novel skeleton from *Leptosphaeria* sp. IV403, an endophytic fungus in *Artemisia annua*. Helv. Chim. Acta.

[30] Schulz B., Boyle C., Draeger S., Römmert A.K., Krohn K. (2002). Endophytic fungi: a source of novel biologically active secondary metabolites. Mycol. Res..

[31] Liu C.H., Zou W.X., Lu H., Tan R.X. (2001). Antifungal activity of *Artemisia annua* endophyte cultures against some phytopathogenic fungi. J. Biotechnol..

[32] Liu C.H., Liu J.Y., Huang L.L., Zou W.X., Tan R.X. (2003). Absolute configuration of keisslone, a new antimicrobial metabolite from *Keissleriella sp*. YS4108, a marine filamentous fungus. Planta Med..

[33] Gary A.S. (2003). Endophytes as sources of bioactive products. Microbes Infect..

[34] Wiyakrutta S., Sriubolmas N., Panphut W., Thongon N. (2004). Endophytic fungi with anti-microbial, anti-cancer and anti-malarial activities isolated from Thai medicinal plants. World J. Microbiol. Biotechnol..

[35] Gao Q., Cao T. (2000). Bryophyta: Hepaticae, Anthocerotae. Flora Yunnanica.

[36] Schulz B., Sucker J., Aust H.J., Krohn K., Ludewig K., Jones P.G., Doring D. (1995). Biologically active secondary metabolites of endophytic Pezicula species. Mycol. Res..

